# Role of NADPH oxidase-2 in the progression of the inflammatory response secondary to striatum excitotoxic damage

**DOI:** 10.1186/s12974-019-1478-4

**Published:** 2019-04-17

**Authors:** Diego Rolando Hernández-Espinosa, Lourdes Massieu, Teresa Montiel, Julio Morán

**Affiliations:** Instituto de Fisiología Celular, División de Neurociencias, Universidad Nacional Autónoma de México (UNAM), Apartado Postal 70-253, 04510 Ciudad de México, Mexico

**Keywords:** NADPH oxidase, NOX-2, Glutamate, Excitotoxicity, Striatum, Caspase-3, Neuroinflammation, Interleukin-4, Interleukin-10

## Abstract

**Background:**

During excitotoxic damage, neuronal death results from the increase in intracellular calcium, the induction of oxidative stress, and a subsequent inflammatory response. NADPH oxidases (NOX) are relevant sources of reactive oxygen species (ROS) during excitotoxic damage. NADPH oxidase-2 (NOX-2) has been particularly related to neuronal damage and death, as well as to the resolution of the subsequent inflammatory response. As ROS are crucial components of the regulation of inflammatory response, in this work, we evaluated the role of NOX-2 in the progression of inflammation resulting from glutamate-induced excitotoxic damage of the striatum in an in vivo model.

**Methods:**

The striata of wild-type C57BL/6 J and NOX-2 KO mice (gp91^Cybbtm1Din/J^) were stereotactically injected with monosodium glutamate either alone or in combination with IL-4 or IL-10. The damage was evaluated in histological sections stained with cresyl violet and Fluoro-Jade B. The enzymatic activity of caspase-3 and NOX were also measured. Additionally, the cytokine profile was identified by ELISA and motor activity was verified by the tests of the cylinder, the adhesive tape removal, and the inverted grid.

**Results:**

Our results show a neuroprotective effect in mice with a genetic inhibition of NOX-2, which is partially due to a differential response to excitotoxic damage, characterized by the production of anti-inflammatory cytokines. In NOX-2 KO animals, the excitotoxic condition increased the production of interleukin-4, which could contribute to the production of interleukin-10 that decreased neuronal apoptotic death and the magnitude of striatal injury. Treatment with interleukin-4 and interleukin-10 protected from excitotoxic damage in wild-type animals.

**Conclusions:**

The release of proinflammatory cytokines during the excitotoxic event promotes an additional apoptotic death of neurons that survived the initial damage. During the subsequent inflammatory response to excitotoxic damage, ROS generated by NOX-2 play a decisive role in the extension of the lesion and consequently in the severity of the functional compromise, probably by regulating the anti-inflammatory cytokines production.

**Electronic supplementary material:**

The online version of this article (10.1186/s12974-019-1478-4) contains supplementary material, which is available to authorized users.

## Background

During excitotoxic damage, neuronal death produced by the overstimulation of the glutamate receptors is determined by several factors, including the increase of intracellular calcium [1], reactive oxygen species (ROS) production [2], microglia activation, and inflammatory responses [3]. Among these factors, the increase in ROS contributes significantly to the subsequent inflammatory process [4]. This condition has implications not only at the onset of damage, but also during the progression of the neuronal death [5].

Although the mitochondria have been classically considered as the main source of ROS [6], the relevance of other sources of ROS such as the NADPH oxidases family (NOX), which are widely distributed in all the studied species and are present in basically all the cell types evaluated, has gained great importance as mediators of excitotoxic damage [7]. In this regard, the genetic or pharmacological inhibition of NOX activity constitutes a neuroprotective condition in several pathologies involving excitotoxicity, such as hypoxia, hypoglycemia, and neuroinflammation [8–10]. Among the homologs of the NOX family, NOX-2 seems to be the main responsible for the observed neuronal death in numerous models of excitotoxic damage [11, 12]. The specific inhibition of NOX-2 in neurons, astrocytes, and microglia prevents neuronal death in different experimental conditions [13, 14]. In a previous study, we observed in genetically deficient NOX-2 mice and wild-type animals treated with a NOX inhibitor that ROS production, lesion size, and microglial reactivity were markedly decreased during excitotoxic damage [13].

A large body of evidence points to neuroinflammation as a main component of most of the central nervous system (CNS) disorders, including those involving excitotoxic damage [15]. Local immune cells orchestrate a series of events in order to stimulate tissue reparation in response to cell damage [16]. Microglia activation constitutes the first line of defense against brain tissue injury [17], through the induction of a range of phenotypes from resting ramified cells to motile amoeboid microglial cells [18]. At the molecular level, a variety of pro- and anti-inflammatory mediators, including interleukins, as well as ROS, nitric oxide, and eicosanoids are produced [19]. In contrast to proinflammatory mediators that participate in neuronal death, the anti-inflammatory mediators can contribute to neuroprotective actions [20, 21].

Cytokines are pleiotropic molecules that define the characteristics and the resolution of the immune response [22]. The characteristics of the response are subordinated to the sum of the proinflammatory and anti-inflammatory stimuli in the microenvironment. TNF-α, IL-1β, IL-6, IL-8, and IL-12, among others, are considered as classic proinflammatory molecules [23–25], while TGF-β, IL-4, and IL-10 are classified as anti-inflammatory cytokines [26, 27].

A simplified classification of the polarization of microglia has been developed, and two phenotypes were proposed based on functional characteristics, the type of cytokines released, and the presence of some molecular markers [28]. The M1 phenotype refers to the classically activated macrophages, which is characterized by the expression of proinflammatory cytokines [29] and the M2 phenotype designates the alternatively activated macrophages that are related to the production of anti-inflammatory cytokines and the resolution of the process [30–32].

Although the M1/M2 concept is widely accepted, it should be noted that the new technologies and the recent data in the field have led to reconsider this classification. For example, both “M1” and “M2” markers can be simultaneously expressed in the same microglial cells or macrophages [33] and M2 cells show a wide range of functional and biochemical features [34]. Thus, the microglial/macrophage polarization seems to be a more complex process than originally described and the M1/M2 phenotypic classification has been reviewed [35, 36], and different intermediate phenotypes have been described on the basis of their functionality and the molecular markers expressed under different physiological states [35, 37].

The mechanisms mediating the inflammatory response of macrophages/microglia are not completely understood, but evidence has provided a framework to propose that ROS may shape the inflammatory response [38, 39], as well as the regulation of microglial activation [40, 41]. This is in line with the association between ROS and several microglia-driven neuropathologies [38]. On the other hand, experimental evidence suggests that microglial NOX2 is involved in neurotoxicity by both producing extracellular ROS [41] and by participating in the redox signal involved in the inflammatory response [42, 43]. For example, NOX activation by interferon and LPS results in microglial expression of iNOS and cytokines mediated by the activation of MAP kinases, NFκB, and STAT1 phosphorylation [44, 45].

It has been suggested that NOX2 plays a key role in the expression of the M1 phenotype by a mechanism mediated by IL10 and STAT3 [37, 46]. Furthermore, the lack of NOX2 leads to a M2 phenotype mediated by a downregulation of NFκB nuclear translocation in a model of TBI and diabetes [46, 47]. Thus, ROS derived from glial cells and from different sources, including NOX2, participate in the microglial activation and play a key action by defining the microglial phenotype, which may have a central role in neurotoxicity and neurodegenerative diseases [43, 45].

In this study, we evaluated the time course of cell damage, NOX activity, and production of cytokines, as well as the functional sequelae, resulting from the excitotoxic damage in an in vivo model. We also tested the protective action of two exogenous anti-inflammatory cytokines in this model. We propose that NOX-2, besides participating in the neuronal excitotoxic damage, also regulate the production of pro- and anti-inflammatory cytokines, which results critical for the progression of the cell death.

## Methods

### Animals

All animals were handled according to the National Institutes of Health Guide for the Care and Use of laboratory animals (NIH Publication No. 8023, revised 1978) and the local Committee for the Care and Use of Laboratory Animals (CICUAL protocol JMA72-15). All efforts were made to minimize pain, as well as the number of animals used. Wild-type (WT) adult mice C57BL/6 (8 weeks old) were obtained from the bioterium of the Instituto de Fisiología Celular, Universidad Nacional Autónoma de México. The NOX-2 KO (gp91^Cybbtm1Din/J^) adult mice generated on a C57BL/6 background were purchased from the Jackson Laboratory (Bar Harbor, ME), and the colony was established at the vivarium of the Instituto de Fisiología Celular, Universidad Nacional Autónoma de México. Mice were housed and kept under controlled temperature (20–22 °C) with a regulated 12-h light-dark cycle, with water and food ad libitum. The mice used for this study were divided as follows: 40 animals were used for histological analyses, 30 for immunoblot and enzyme activity determinations (NOX and caspase-3), and 30 for cytokine analysis.

### Intrastriatal glutamate injection

**Fig. 1:**
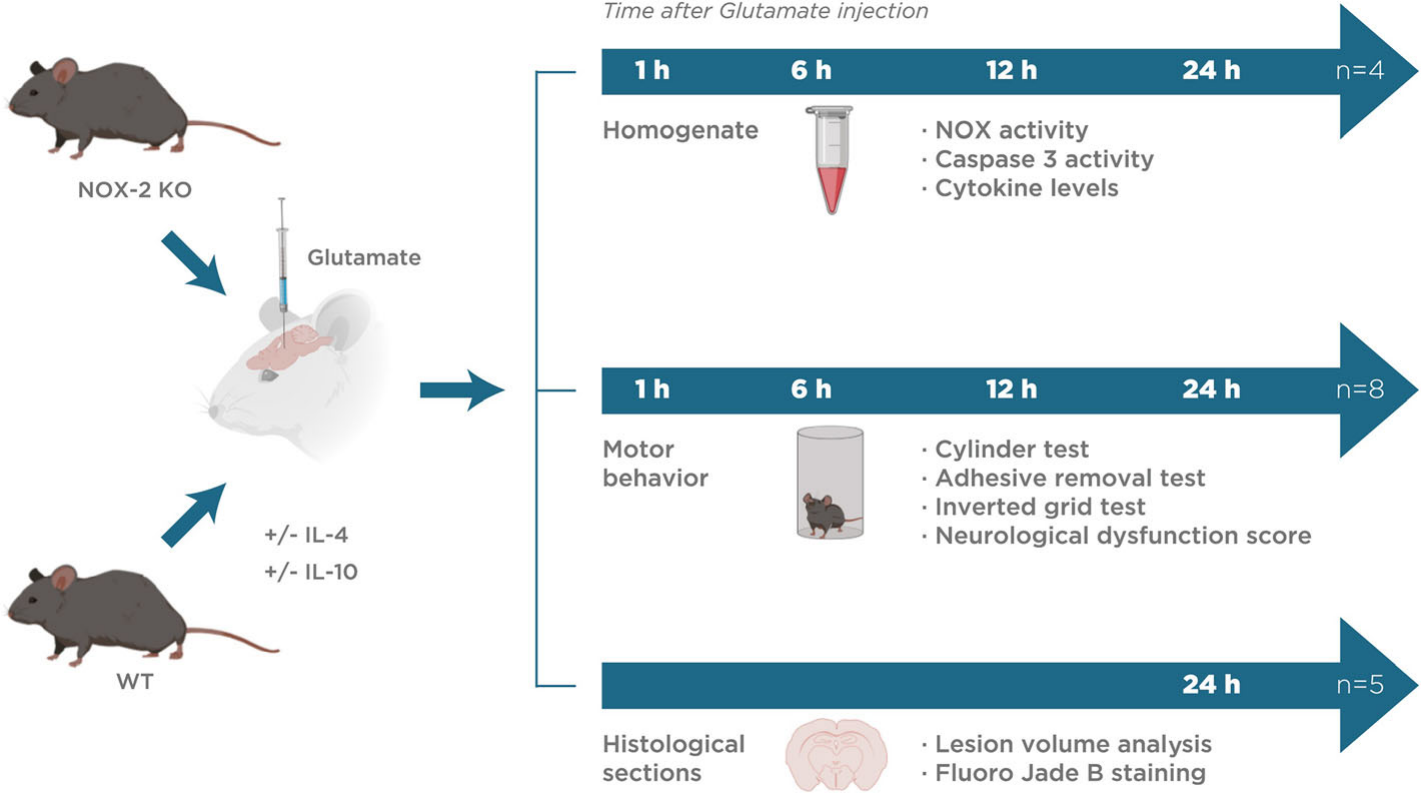
Illustration of the timeline experimental design. Wild mice (WT, without treatment and treated with interleukins 4 or 10) and NOX-2 KO were administered with monosodium glutamate (1 M). Subsequently, they were sacrificed at 24 h to obtain histological sections (*n* = 5). Some animals were sacrificed at 1, 6, 12, and 24 h to obtain striatum homogenate (*n* = 4). Motor behavior tests were performed at 1, 6, 12, and 24 h in animals of both groups (*n* = 8)

C57BL/6 WT and NOX-2 KO mice were anesthetized with 3% isoflurane in a 95% O2/5% CO2 mixture and placed on a stereotaxic frame (David Kopf Instruments, Tujunga, CA). A stainless steel needle was positioned in the right striatum according to the following coordinates: anterior-posterior + 0.8 mm from bregma, lateral + 2.2 mm from midline, and vertical 3.2 mm from dura [35]. With the aid of an injection pump (model 55; Harvard, South Natick, MA), 0.5 μL of a solution of monosodium glutamate (1 M) or saline solution (0.9%) was injected (0.175 μL/min). A second (25 WT animals) and a third group (25 WT animals) of mice were treated with 0.7 ng/mL of interleukin-4 (cell signaling #5208) or 0.4 ng/mL of interleukin-10 (cell signaling #5261), respectively, in the same monosodium glutamate solution (Fig. 1). Five minutes after the injection, the needle was gently withdrawn, and the skin was sutured with nylon [48]. Mice recovered from anesthesia in a temperature-controlled chamber and were placed in individual cages with water and food ad libitum.

At different times after the surgery, animals received a lethal dose of sodium pentobarbital to subsequently extract the brain. For biochemical analyses, some animals were killed by cervical dislocation at different times (1, 6, 12, and 24 h) and striata were dissected and homogenized. To that, the olfactory bulb and cerebellum were removed from the brain and a sagittal section was made on the midline to separate the hemispheres and dissect the striate, which can be delimited as tissue slightly more transparent than the surrounding cortex [49]. The tissue obtained (15–20 mg) was homogenized in lysis buffer and protease inhibitors at 4 °C (OMNI TC homogenizer). The homogenate was centrifuged at 5000 g for 5 min to remove the cellular debris and subsequently the amount of protein was quantified [50].

### Lesion volume analysis

Glutamate-induced lesions were evaluated 24 h after the surgery (Fig. 1). Mice were deeply anesthetized with sodium pentobarbital and were transcardially perfused with 30 mL of 0.9% saline solution followed by 35 mL of 4% paraformaldehyde solution in 0.1 mM phosphate buffer at pH 7.4. Brains were removed and placed in the same fixative solution at 4 °C. Consecutive series of striatal coronal sections (40 μm thick) for cresyl violet and Fluoro-Jade B (FJB; Chemicon, Temecula, CA) staining were obtained in a cryostat (1510s, Leica, Microsystems Nussloch GmbH, Heidelberger Nussloch, Germany). After staining with cresyl violet, all sections where a lesion was visible (pale cresyl violet stain) were selected for quantification of the lesion volume. The damaged areas were delineated manually and measured using an image analyzer (ImageJ 1.48v analyzer program; Wayne Rasband, NIH, USA) by an experimenter blinded to the treatments. Total lesion volume was obtained from the sum of all damaged areas multiplied by the width of the sections (40 μm).

### Fluoro-Jade B staining

Briefly, one drop of 1% NaOH solution diluted in 80% ethanol was added to coronal sections mounted on slides, and after 2 min, it was replaced by a 70% ethanol solution. Slides were covered with 0.06% potassium permanganate for 10 min, and then brain sections were washed and incubated for 10 min with a 0.0004% Fluoro-Jade B (FJB) solution prepared in 0.1% acetic acid. Finally, the sections were washed, dried at 50 °C, dehydrated with xylol, and covered with Permount (Fisher Scientific, Fair Lawn, NJ). Slices were observed under an epi-fluorescence microscope (Eclipse Ti-S, Nikon instruments Inc.) using a U-MNB2 filter (395–590 nm). Total FJB-positive cells were counted as previously described using the ImageJ.1.48v analyzer program (Wayne Rasband, NIH, USA) in the entire striatum in three sections per mouse, namely, the section containing the needle tract (middle section) and the adjacent anterior and posterior sections located 160 μm away from the middle section [48].

### NOX activity

NOX activity was determined in striatal homogenates at different times (0.5, 1, 3, 6, 12, and 24 h) after glutamate administration (Fig. 1). NOX activity was estimated as the oxidation of dihydroethidium (DHE) to ethidium (Et) [48]. Briefly, tissue homogenates were incubated with 0.02 mM DHE, 0.5 mg/mL salmon DNA, and 0.2 mM NADPH as substrate. Et fluorescence was measured during 30 min at an excitation wavelength of 480 nm and emission of 610 nm using a Synergy HT Multi-Detection fluorescence microplate reader (Biotek Instruments, Colchester, VT). Duplicate samples were prepared, and NOX activity was expressed as the change in Et fluorescence per milligram protein per minute versus control (saline). Some samples were incubated in the presence of 1 μM of the NOX inhibitor diphenyleneiodonium (DPI; Sigma) or 15 UI of superoxide dismutase (SOD, Sigma) to corroborate the specificity of the assay.

### Immunoblot

Tissue homogenates (50 μg protein per lane) were subjected to SDS–PAGE. The resolved proteins were transferred to PVDF membranes at 120 V for 1 h. The membranes were blocked overnight with 5% nonfat dry milk in TBS and treated, overnight at 4 °C, with anti-NOX2/gp91phox (1:500; ab80508, Abcam, USA) and anti-GAPDH (1:3000; 14C10, Cell signaling, USA) antibodies. After further washing, the blots were incubated with alkaline phosphatase-conjugated secondary antibody (1:10000) for 1 h at room temperature. Bands were visualized by using the enhanced chemiluminescence system according to the manufacturer’s recommendations (Bio-Rad Laboratories, Hercules, CA) and exposed to Kodak XAR-5 film.

### Caspase-3 activity

Caspase-3 activity was determined in striatal homogenates at different times (1, 3, 6, 12, and 24 h) after glutamate administration (Fig. 1). Caspase-3 activity was assayed by a fluorogenic technique [51] in a luminescence spectrometer (Shimadzu, RF-5301PC), using the peptide Ac-VDVAD-AMC as a substrate to detect the proteolytic activity. Caspase activity was followed 30 min after addition of substrate (80 μM) and tissue homogenate (50 μg/mL) in a standard solution. Results depicting caspase activity were calculated as the change in fluorescence intensity per milligram protein per minute and expressed as fold change versus control (saline).

### Cytokine levels

Cytokine levels were measured using the mouse ultrasensitive ELISA kit assay for IL-1β (ab100704), IL-4 (ab100710), IL-6 (Abcam, ab100712), IL-10 (ab46103), IL-12 p40/70 (100699), TNF-α (100747), and TGF-β (119557). The tissue samples, corresponding to 100 μg of striatal protein (diluted 1:10), were obtained after different times (1, 3, 6, 12, and 24 h) of glutamate administration (Fig. 1) and were used for cytokines measurement according to the manufacturer’s protocol (Abcam, MA, USA). Assays were carried out in a colorimetric spectrometer (Shimadzu, RF-5301PC). The results were calculated based on a concentration curve provided by the manufacturer and were expressed as picograms of cytokine per milligrams of protein.

### Cylinder test of forelimb asymmetry

Animals were examined for preferential use of the unilateral forelimb during upright postural support before and after glutamate administration (Fig. 1). Briefly, mice were placed in a pyro-glass cylinder (10 cm in diameter) on a tabletop and video-recorded for 3 min. The number of unilateral and bilateral wall contacts was recorded. The percentage of bilateral contacts was assessed on each test using the formula 100 × bilateral contacts/total forelimb wall contacts, whereas the percentage of unilateral contacts was assessed using the formula 100 × unilateral contacts/total forelimb wall contacts. The results are expressed as the percent of unilateral exploration [52].

### Adhesive removal test

The animal cage was placed in the testing room at least 30 min before starting the experiment to allow habituation to the new environment. Animals were gently removed from the testing box and an adhesive tape strip (0.2-in. square) was placed on the snout (dorsal portion). Animals were returned to the testing cage and a timer was set. Conduct was observed and recorded for 60 s or until the piece of tape was removed with the forelimb. Training consisted of five trials (one per day), and testing after glutamate administration at different times (1, 3, 6, 12, and 24 h) was carried out (Fig. 1). Result indicated the latency to adhesive tape removal in seconds [53].

### Inverted grid test

The inverted grid test was used to assess coordination and limbs muscular strength, especially related to distal musculature and digit manipulations. Mice were placed in the center of a horizontal square (15 cm^2^) grid consisting of a wire mesh (0.5 cm^2^) surrounded by wooden walls. The grid was placed 20 cm above a tabletop and was rotated upside down allowing mice to move freely. Each mouse was recorded for 60 s. The time the mice remained and moved upside down was recorded. If a mouse fell from the mesh grid within 10 s, additional trials were allowed (max three trials) within an interval of 1 min, in this case, latencies before falling were measured. The means ± standard error of the mean (SEM) of three trials were calculated. No pretraining was performed, but a pretest of 30 s before the day of the experiment was carried out for habituation [53].

### Neurological dysfunction score

All behavioral tests were conducted by the same experimenter in a quiet and low-lit room with white noise background. Pretests were conducted to exclude abnormally behaving animals. A combination score from a battery of three behavioral tests (adhesive test, inverted grip test, and cylinder test) was used to measure the neurological functional deficits (ND). We determined the grand NDs score (NDS) by combining all the tests, assuming an equal weight of each of the tests and assigning a value of 15 points to the maximum performance [53, 54].

### Statistical analysis

Data were analyzed as means ± SEM. The data were evaluated statistically by the two-way analysis of variance (ANOVA) for biochemical and histological tests and repeated measures ANOVA for behavioral tests, followed by Tukey’s test for pairwise comparisons by using GraphPad Prism v6.0 and SigmaPlot 12.3 software. In the case of significance, a further ad hoc two-tailed Student’s *t* test was applied. Significance was considered when *p* < 0.05.

## Results

### Striatal excitotoxic lesion and neuronal degeneration are minor in NOX-2 KO mice

**Fig. 2:**
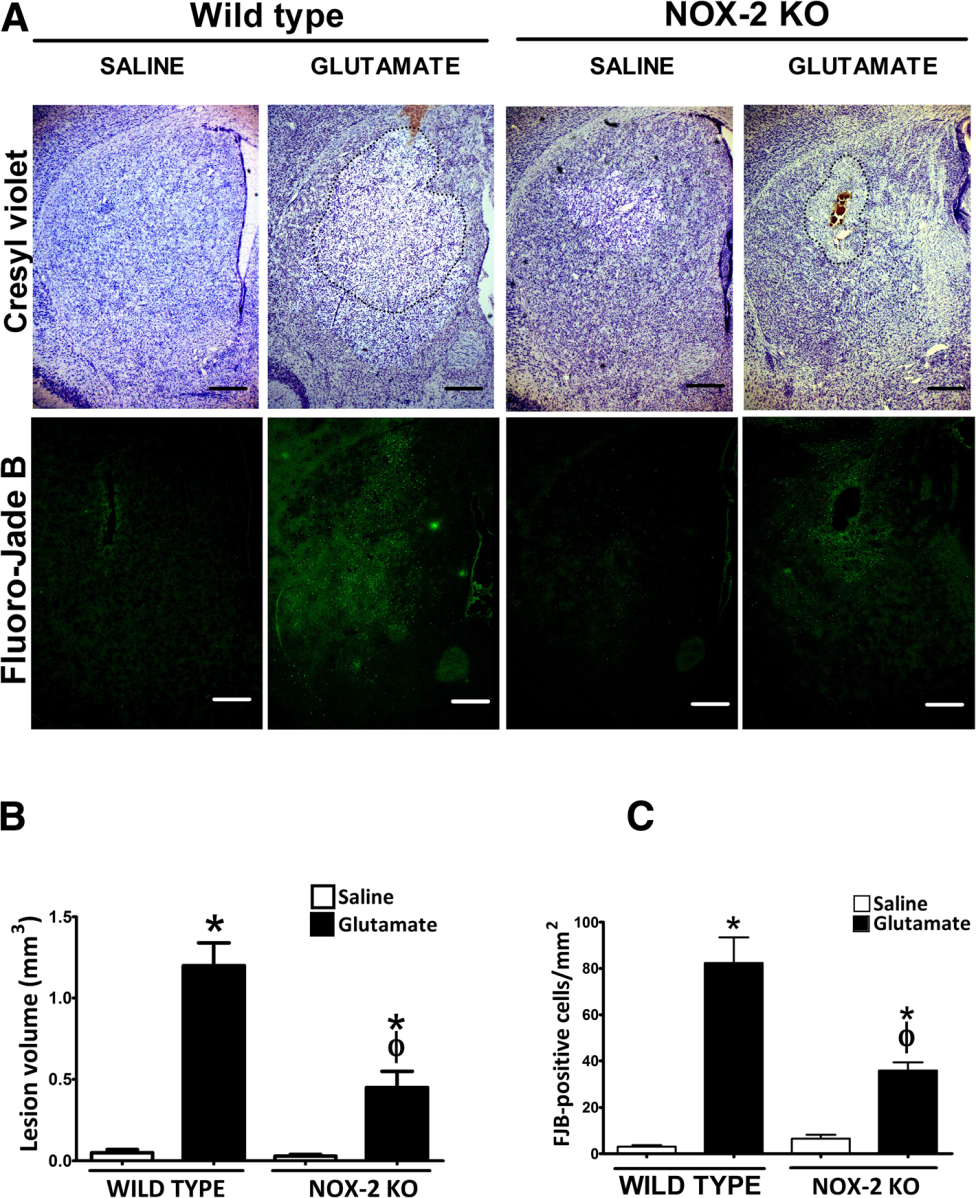
Lesion area and neuronal degeneration in wild-type and NOX-2 KO mice injected with glutamate in the striatum. Histological sections were obtained after 24 h of intracerebral administration of saline (NaCl 0.9%) or glutamate (1 M) as detailed in the “Methods” section. **a** Representative micrographs of coronal striatal sections showing striatal lesions stained with cresyl violet and damaged cells positive to Fluoro-Jade B. The dotted line delimits the area of lesion of mice treated with glutamate. The scale bars represent 200 μm. **b** Quantification of the lesion volume 24 h after saline or glutamate injection is expressed in cubic millimeters. **c** Total number of FJB-positive cells counted in three slices per mice. Data are expressed as means ± SEM of five independent experiments. *p < 0.05 vs the corresponding saline control; ^ϕ^*p* < 0.05 vs wild-type mouse (WT) treated with glutamate

To determine the impact of excitotoxic damage, both lesion volume and neuronal degeneration were evaluated in WT and NOX-2 KO mice as detailed in the “Methods” section. Representative tissue sections from WT and NOX-2 KO animals stained with cresyl violet are shown in Fig. 2a. WT and NOX2 KO mice treatment with saline, as a control of the procedure, developed a small lesion similar in both groups (0.05 ± 0.02 mm^3^ and 0.03 ± 0.01 mm^3^, respectively). As it was expected, in the groups treated with glutamate, NOX-2 KO mice showed only 38% of the lesion observed in WT mice (WT, 1.2 ± 0.14 mm^3^ vs NOX-2 KO, 0.45 ± 0.10 mm^3^ *p* < 0.05; Fig. 2b).

The FJB-positive cells were counted in tissue sections 24 h after glutamate injection (Fig. 2c). Again, a higher number of degenerated cells were found in WT mice injected with glutamate (82.3 ± 11.1 cells/mm^2^) than in saline-injected animals (3.06 ± 0.6 cells/mm^2^; *p* < 0.05). In accordance to the tissue lesion measurements, the number of FJB-positive cells in NOX-2 KO mice (35.9 ± 7.5 cells/mm^2^; *p* < 0.05) were about half of that observed in WT mice treated with glutamate. Representative tissue sections from WT and NOX-2 KO animals showing FJB-labeled cells are shown in Fig. 2a. Together, these results confirm that NOX-2 participates in the tissue injury and neuronal degeneration induced by glutamate.

### NOX-2 activity increases during excitotoxic damage

**Fig. 3:**
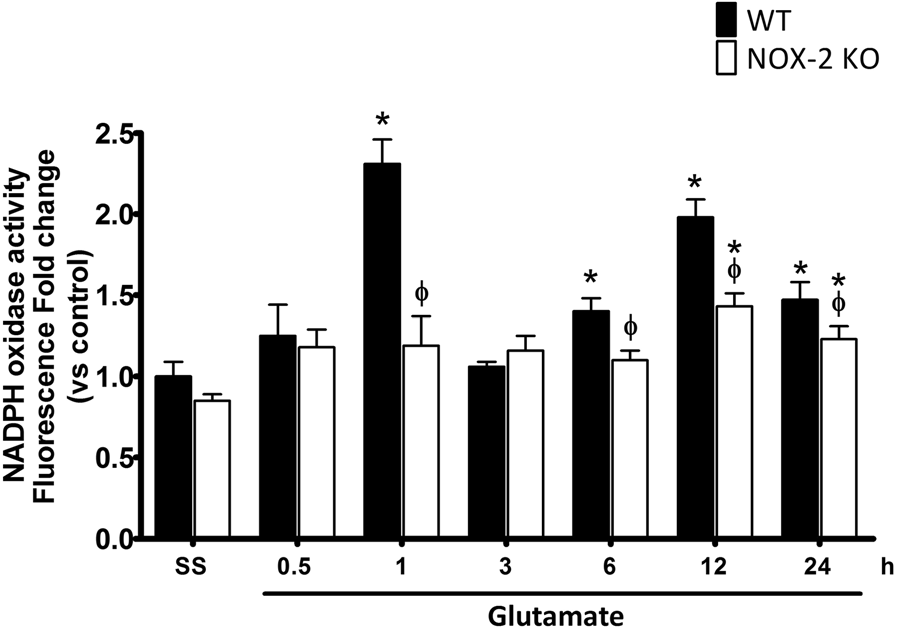
NOX-2 activity during excitotoxic damage in wild-type and NOX-2 KO mice. NOX activity was evaluated as the change of fluorescence intensity resulting from dihydroethidium (DHE) oxidation to ethidium (Et) in striatum homogenates of WT and NOX-2 KO mice treated with glutamate (1 M) 0.5, 1, 3, 6, 12, and 24 h after glutamate administration. Data are expressed as fold change of Et fluorescence relative to WT saline. Values are means ± SEM of four independent experiments. *p < 0.05 vs the corresponding saline control; ^ϕ^*p* < 0.05 vs the corresponding wild-type mouse (WT) treated with glutamate

In the present study, we measured NOX activity in striatal homogenates of WT and NOX-2 KO mice at different times after glutamate injection (0.5, 1, 3, 6, 12, and 24 h). An increase in the enzyme activity over saline (control) levels was detected at 1 h after glutamate administration in WT mice (225 ± 27% vs control, *p* < 0.05; Fig. 3). Although the NOX activity remained increased during 24 h, we observed two peaks of activation, the first 1 h after glutamate injection (130 ± 15% vs control) and the second after 12 h (195 ± 10% vs control). The increase of NOX activity in NOX-2 KO mice after glutamate administration was more discrete and about half of that in WT mice at 12 and 24 h (*p* < 0.05). These results indicate that the administration of glutamate leads to an early and a late activation of NOX during the excitotoxic damage and that this activation is attenuated in NOX-2 KO animals.

We found that NOX2 protein was upregulated by glutamate treatment in a time-dependent manner. The protein levels increased from 6 h and continued increasing after 12 and 24 h (Additional file 1: Figure S1). The observed changes in protein levels did not correlate with the observed changes in NOX activity. For example, at 1 h no change in the protein was observed, while a large increase in NOX activity occurred (Fig. 3 and Additional file 1: Figure S1). This suggests that the observed changes in NOX activity are regulated by other conditions than protein enrichment.

### NOX-2 KO mice resistance to excitotoxic damage involves a decrease in caspase-3 activation

**Fig. 4:**
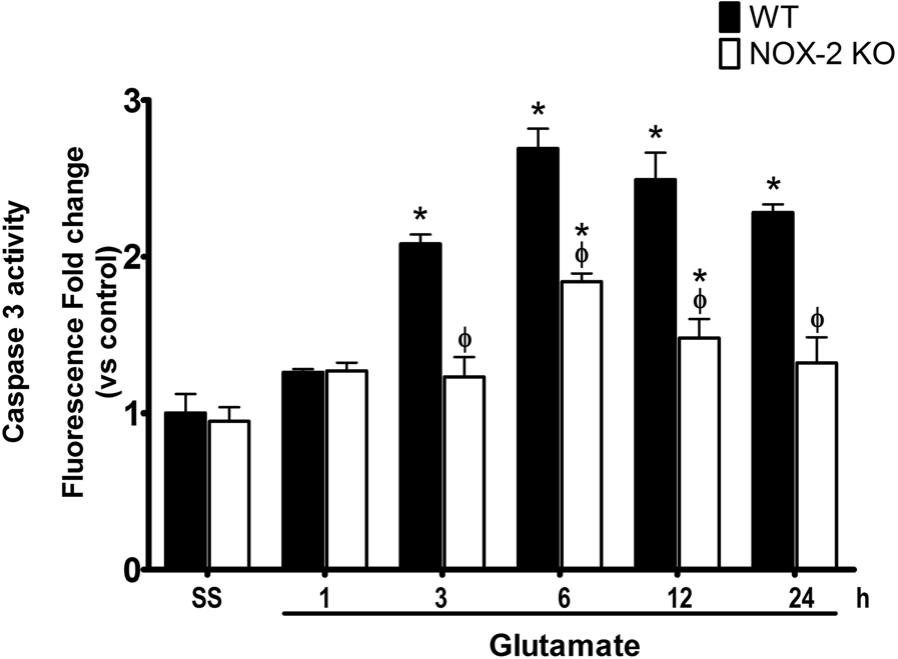
Caspase-3 activity in wild-type mice and NOX-2 KO after administration of glutamate. Active caspase-3 was measured in striatum homogenates after 1, 3, 6, 12, and 24 h of glutamate (1 M) administration in WT and NOX-2 KO mice. Fluorescence produced by the cleavage of the specific substrate for caspase-3 was quantified in relative units of fluorescence (FRU) and the results are expressed as the fluorescence fold change relative to WT saline. Values are mean ± SEM of three independent experiments. *p < 0.05 vs the corresponding saline control; ^ϕ^*p* < 0.05 vs corresponding wild-type mouse (WT) treated with glutamate

Intrastriatal injection of glutamate causes an increase of caspase-3 activation at 3 h after glutamate administration in WT (208 ± 9% vs control) and after 6 h in NOX-2 KO mice (197 ± 16% vs control). We found that after 6 h, caspase-3 activation was lower in NOX-2 KO (*p* < 0.05) as compared to WT mice (NOX-2 KO 180 ± 10% vs WT 280 ± 25%, *p* < 0.05), and the same effect is observed during the following 24 h (NOX-2 KO 57 ± 15% vs WT 128 ± 9%, *p* < 0.05; Fig. 4). Thus, NOX-2 KO mice exhibit a lower activation of caspase-3 during excitotoxic injury induced by the administration of glutamate.

### NOX-2 KO mice exhibit better motor recovery than wild-type mice after excitotoxic damage

**Fig. 5:**
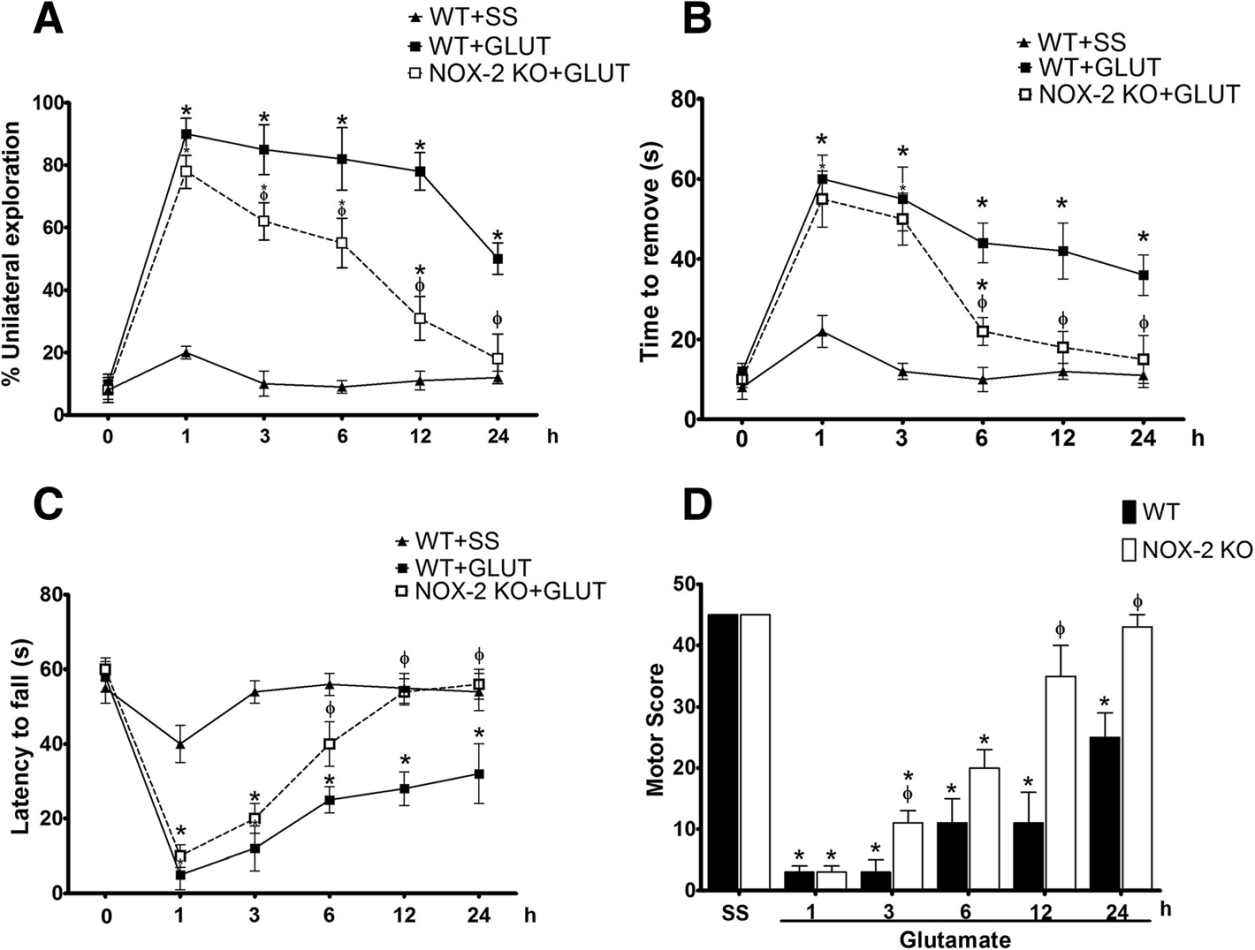
Motor activity recovery after glutamate administration in NOX-2 KO mice. Performance of wild-type (WT + SS) animals and WT or NOX-2 KO mice injected with glutamate (WT + GLUT; NOX-2 KO + GLUT) was evaluated in three tests of motor behavior at 1, 3, 6, 12, and 24 h after glutamate intracerebral injection. These tests were later integrated to obtain a motor score that reflects functional deterioration. **a** Cylinder test in which each mouse obtained a percentage based on the ratio of unilateral and bilateral explorations performed in three trials, of 3 min each; the percentage was expressed as mean ± SEM of eight independent tests. **b** Adhesive removal test that quantifies the time in which the animals remove a label previously placed by the examiner. The time of removal was quantified in seconds and was expressed as means ± SEM of six independent animals. **c** The tone and muscle strength of the four limbs was evaluated by exposing the mice to the inverted grid test in which the decay time was quantified for three non-consecutive opportunities of a maximum of 60 s of duration. The results are expressed as means ± SEM of the latency to fall of six independent tests. **d** To integrate the three previous tests, an arbitrary score was assigned to the performance of each animal, giving the highest score to the performance of the mice injected with saline (45 points). The results are expressed in arbitrary units of motor score as the mean ± SEM of the latency to fall of six independent tests. *p < 0.05 vs the corresponding saline control; ^ϕ^*p* < 0.05 vs the corresponding wild-type (WT) mouse treated with glutamate

Striatum lesions lead to alterations in the control of voluntary movements and muscle tone, predominantly in the forelimbs [53]. We therefore explored the degree of functional compromise resulting from the excitotoxic damage as an index of striatal injury. To assign a neurological deficit score (NDS), we evaluated three tests in WT and NOX-2 KO mice to obtain a motor score (Fig. 5).

The cylinder test indicates the latency of forelimb asymmetry (Fig. 5a). Prior to glutamate administration, WT and NOX-2 KO mice tended to preferentially make bilateral wall contacts in the cylinder before glutamate administration (WT 10 ± 3% and NOX-2 KO 8 ± 2% of unilateral contacts). Excitotoxic damage in WT mice resulted in an early and sustained decrease in bilateral contacts from 1 h (90 ± 5% unilateral contacts) to 24 h (50 ± 4% unilateral contacts). NOX-2 KO mice show a similar compromised motor function that WT mice at 1 h (78 ± 6% unilateral contacts; *p* < 0.05), but despite the fact that they exhibited a severe decline at short time, they managed to recover at 24 h (18 ± 8% unilateral contacts), unlike the WT, which do not reach control levels (*p* > 0.05; Fig. 5a).

The adhesive removal test evaluates the control of voluntary movements (Fig. 5b). Prior to administration of glutamate, both WT and NOX-2 KO mice took a maximum of 14 s to remove the adhesive (WT 12 ± 2 s and NOX-2 KO 10 ± 2 s). After 1 h of glutamate administration, WT mice were unable to remove the label before 60 s. After 24 h, WT mice showed an improvement in the ability to remove the label; however, the time to removal was markedly higher than that shown by the saline controls (WT + GLU 37 ± 5 s vs WT + SS 11 ± 3 s, *p* < 0.05). NOX-2 KO mice had a similar performance to that shown by the WT mice after 1 h (WT 55 ± 5 s vs NOX-2 KO 50 ± 7 s, *p* > 0.05). However, at 6 h, the NOX-2 KO mice showed a marked recovery as compared to WT mice (WT 44 ± 5 s vs NOX-2 KO 22 ± 4 s; *p* < 0.05). It reaches control levels after 12 h, a situation that did not occur in WT mice (WT 37 ± 5 s vs NOX-2 KO 12 ± 3 s, *p* < 0.05; Fig. 5b).

Finally, the inverted grid test indirectly allows evaluation of the muscle tone of the mice extremities (Fig. 5c). Control mice remained attached to the inverted grid for about a minute (56 ± 4 s), which is considered an adequate performance and is established as the baseline. After 1 hour of glutamate administration, both the WT and NOX-2 KO mice were unable to remain attached for long time to the grid (WT 5 ± 4 s and NOX-2 KO 10 ± 3 s). In comparison to WT mice, the NOX-2 KO mice showed a better functional recovery from 6 h after the event (WT 25 ± 4 vs NOX-2 KO 40 ± 6 s *p* < 0.05) reaching a performance similar to control animals from 12 h, which is not observed in WT mice (WT 32 ± 8 vs NOX-2 KO 54 ± 4 s *p* > 0.05; Fig. 5c).

Motor function parameters were integrated into a NDS (Fig. 5d). To that, each test was given an equivalent weight in the score, assigning an arbitrary value of 15 points (for each test) to the control animals treated with saline solution. Therefore, a poor performance during the test corresponded to a decrease in the points awarded [55]. The WT mice treated with glutamate showed a poor score in the first 3 h (3 ± 1 points), which improved at 12 h (11 ± 5 points) and 24 h (25 ± 4 points). NOX-2 KO mice treated with glutamate obtained a poor score in the first hour (3 ± 1 points), reaching a marked improvement at 24 h with a performance similar to the control animals (43 ± 2 points, *p* > 0.05; Fig. 5d). As we observed in the previous tests, the functional recovery of the NOX-2 KO mice surpasses that observed in WT mice after 12 h of glutamate administration (WT 11 ± 5 vs NOX-2 KO 35 ± 4 s *p* < 0.05). Based on these findings, we suggest that NOX-2 activity is associated with a worst functional motor recovery after glutamate administration.

### The pattern of cytokine production in response to excitotoxic damage depends on NOX-2

**Fig. 6:**
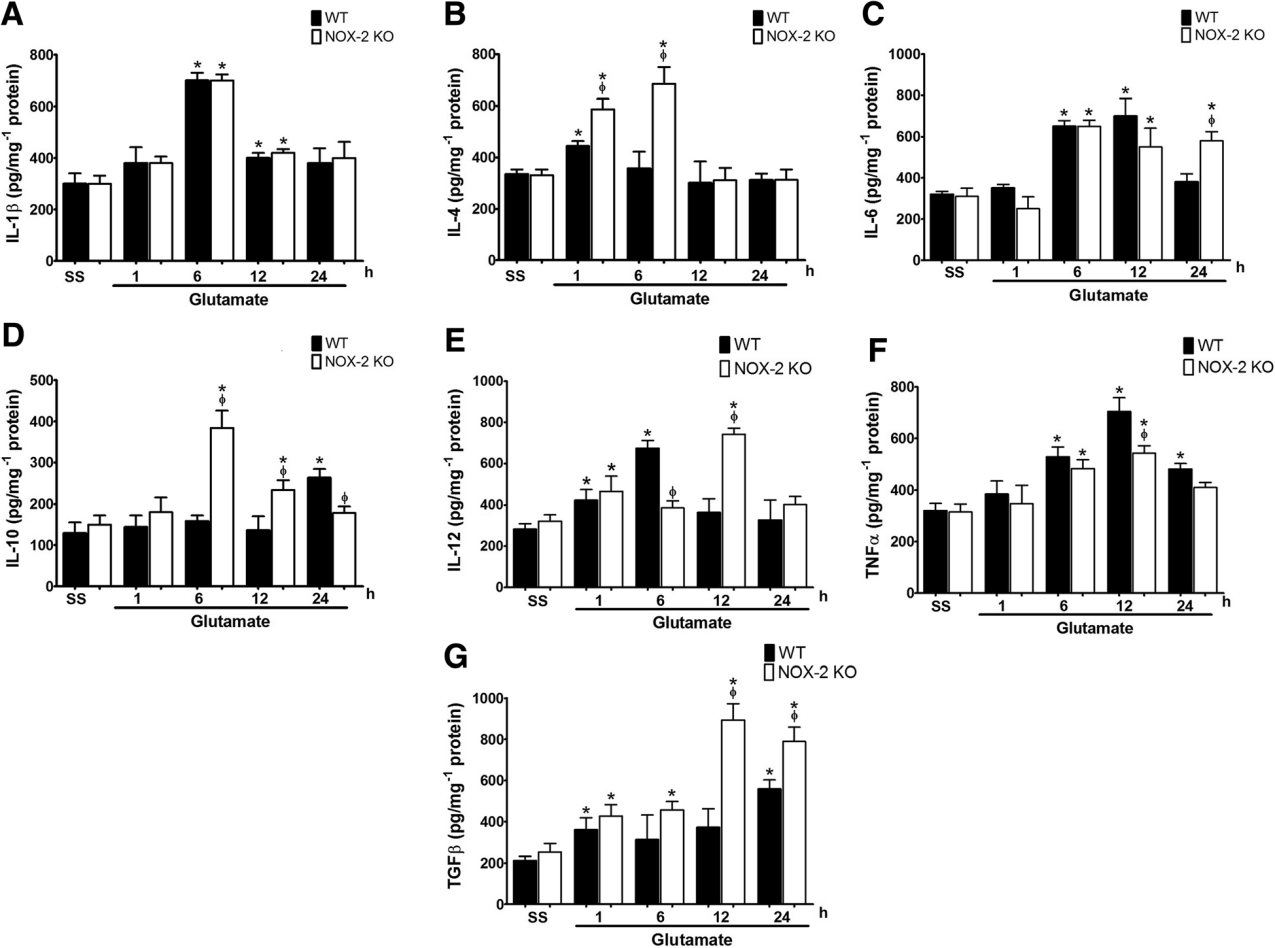
Pattern of cytokine production in wild-type and NOX-2 KO mice after the striatal injection of glutamate. Characterization of the inflammatory response was carried out by determination of cytokines concentration in striatum homogenates of wild-type (WT) and NOX-2 KO mice determined by enzyme-linked immunosorbent assays (ELISA) at 1, 6, 12, and 24 h after administration of saline (SS) or glutamate (1 M) as detailed in the “Methods” section. **a** Interleukin-1β (IL-1β), **b** interleukin-4 (IL-4), **c** interleukin-6 (IL-6), **d** interleukin-10 (IL-10), **e** interleukin-12 (IL-12), **f** tumor necrosis factor α (TNF-α), and **g** transforming growth factor β (TGF-β). Data are expressed as picograms per milligram of protein and are means ± SEM of three independent experiments. *p < 0.05 vs the corresponding saline control; ^ϕ^*p* < 0.05 vs the corresponding WT mouse treated with glutamate

The resolution of the excitotoxic damage depends on the inflammatory response, which aims to remove damaged tissue and recover functions [56]. We characterized the inflammatory response to excitotoxic damage and evaluated the involvement of NOX-2 in the production of cytokines associated with the inflammatory response in the CNS (Fig. 6a).

Regarding the inflammatory cytokines, we observed an increase of IL-1β in the WT (701 ± 30 pg/mg^− 1^ protein) after 6 h of glutamate administration, when compared with the saline control (298 ± 41 pg/mg^−1^ protein). In a similar manner, the NOX-2 KO mice depicted and increased value for this interleukin (682 ± 24 pg/mg^−1^ protein). At 12 h, the IL-1β levels were markedly reduced in both conditions, but still above the control (WT + GLU 400.7 ± 22 vs. NOX-2 KO + GLU 420 ± 15 pg/mg^− 1^ protein, *p* < 0.05), returning to control levels at 24 h (WT + GLU, 380 ± 58 and NOX-2 KO + GLU, 401 ± 63 pg/mg protein; Fig. 6a). No differences were found between WT mice and NOX-2 KO in the production of IL-1β.

Excitotoxic damage also caused a similar increase of the inflammatory cytokine IL-6 in both groups at 6 h and 12 h (WT + GLU, 650 ± 85 and NOX-2 KO + GLU, 553 ± 91 pg/mg^−1^ protein). Unexpectedly, after 24 h of glutamate administration, NOX-2 KO maintained high levels of IL-6 compared to WT mice (WT 381 ± 41 vs NOX-2 KO 581 ± 43 pg /mg^−1^ protein, *p* < 0.05; Fig. 6c).

IL-12 levels increased at the first hour after glutamate treatment in both WT (423 ± 52 pg /mg^−1^ protein) and NOX-2 KO (475 ± 74 pg /mg^−1^ protein) mice as compared to the saline control (WT 282 ± 28) and saline NOX-2 KO (321 ± 31 pg /mg^−1^ protein, *p* < 0.05), respectively. After 6 h, WT mice treated with glutamate showed a maximum increase, which was significantly higher than that observed in NOX-2 KO mice (WT 673 ± 39 vs NOX-2 KO 385 ± 35 pg /mg^−1^ protein, *p* < 0.05). At 12 h, this pattern was inverse since IL-12 levels were much higher in NOX-2 KO than in WT mice (WT 363 ± 67 vs NOX-2 KO 743 ± 29 pg /mg^−1^ protein, *p* < 0.05; Fig. 6e).

We observed an increase of TNF-α induced by glutamate in both WT (528 ± 39 pg /mg^−1^ protein) and NOX-2 KO mice at 6 h (483 ± 35 pg /mg^−1^ protein) and 12 h (WT 704 ± 54; NOX-2 KO 542 ± 29 pg /mg^−1^ protein). The production of TNF-α in NOX-2 KO mice was always lower than in WT, particularly at 6 and 12 h. After 24 h, the observed increase was reduced in both WT (482 ± 22 pg/mg protein) and NOX-2 KO mice (410 ± 20 pg/mg^−1^ protein, *p* > 0.05; Fig. 6f).

Regarding the production of anti-inflammatory cytokines, we measured the concentration of some of the anti-inflammatory cytokines previously reported in the CNS, such as IL-4, IL-10, and TGF-β [21, 22]. One hour after glutamate administration, NOX-2 KO mice showed an almost twofold increase in the production of IL-4 as compared to control (saline) (NOX-2 KO 586 ± 42 vs NOX-2 KO + SS 330 ± 32 pg/mg^−1^ protein; *p* < 0.05), while in WT mice, glutamate induced an increase of 125% with respect to the saline control (WT + GLU 443 ± 20 vs WT + SS 335 ± 18 pg/mg^−1^ protein; *p* < 0.05). The observed difference between WT and NOX-2 KO was markedly increased at 6 h (WT 357 ± 54 vs NOX-2 KO 685 ± 65 pg/mg protein, *p* < 0.05; Fig. 6b). At 12 and 24, no difference was observed between groups and values returned to control values.

The administration of glutamate to NOX-2 KO mice caused an increase of IL-10 more than double respect to WT mice at 6 h (WT 158 ± 14 vs NOX-2 KO 384 ± 42 pg/mg^−1^ protein, *p* < 0.05) and this increase was maintained only in NOX-2 KO group until 12 h (WT 136 ± 34 vs NOX-2 KO 234 ± 24 pg/mg^−1^ protein, *p* < 0.05). However, at 24 h, we observe an increase of IL-10 in WT, while values in NOX-2 KO continued decreasing (WT 263 ± 21 vs NOX-2 KO 178 ± 18 pg/mg^−1^ protein, *p* < 0.05; Fig. 6d).

Regarding the production of TGF-β (Fig. 6g), a negative regulatory cytokine, we observed a similar increase both in WT as NOX-2 KO mice treated with glutamate at 1 h (WT 360 ± 59 vs NOX-2 KO 427 ± 56 pg/mg^−1^ protein, *p* < 0.05). This increase was maintained only in the NOX-2 KO group, until 24 h, reaching a maximum concentration at 12 h (WT 372 ± 91 vs NOX-2 KO 893 ± 79 pg/mg^−1^ protein, *p* < 0.05). At this time, it reaches more than double than that found in WT mice. In contrast, WT mice showed only a second increase at 24 h, which was lower than that observed in NOX-2 KO (WT 557 ± 45 vs NOX-2 KO 789 ± 69 pg/mg^−1^ protein, *p* < 0.05). These results suggest that the change in the cytokines profile in NOX-2 KO mice is characterized by an increase in the production of anti-inflammatory cytokines and that this increase occurs preferentially at early times during the excitotoxic event.

### Administration of IL-4 partially prevents the excitotoxic lesion

**Fig. 7:**
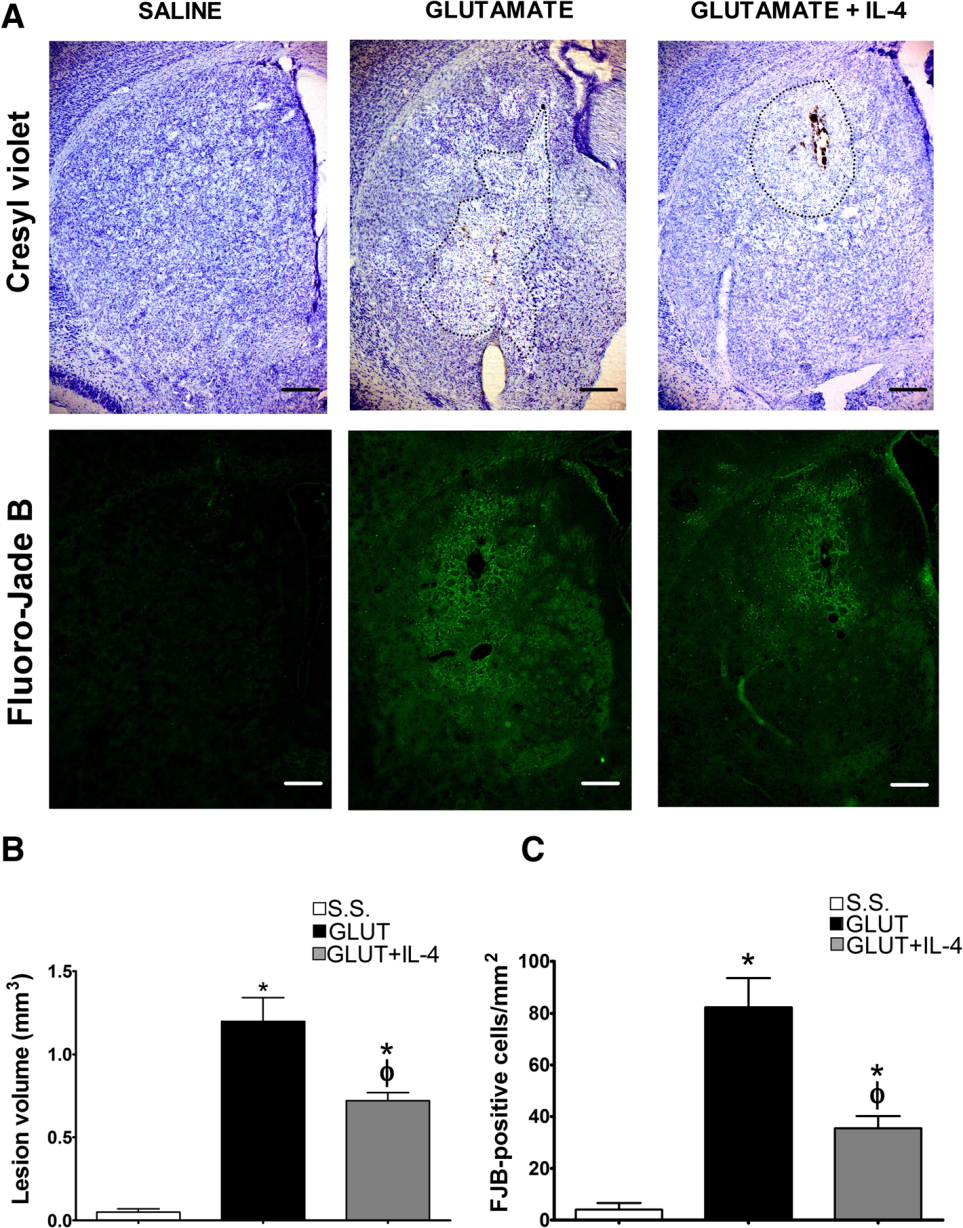
Effect IL-4 on the striatal lesion and neuronal degeneration in wild-type mice treated with glutamate. Histological sections of wild-type (WT) mice were obtained after 24 h of intracerebral administration of NaCl 0.9% (S.S.), only glutamate (GLUT) or glutamate + 0.7 ng/mL of IL-4 (GLUT + IL4) as detailed in the “Methods” section. **a** Representative micrographs of coronal striatal sections showing lesions stained with cresyl violet and damaged cells positive to Fluoro-Jade B. The dotted line delimits the area of lesion of mice treated with glutamate. The scale bars represent 200 μm. **b** Quantification of the lesion volume 24 h after saline or glutamate injection is expressed in cubic millimeters. **c** Total number of FJB-positive cells were counted in three slices per mice. Data are expressed as mean ± SEM of six independent experiments. *p < 0.05 vs saline control (S.S.); ^ϕ^*p* < 0.05 vs the corresponding mouse treated only with glutamate (GLUT)

One of the most evident differences in the response of the NOX-2 KO mice to the excitotoxic damage was the increase in the production of anti-inflammatory cytokines; therefore, we treated animals with exogenous IL-4 or IL-10 simultaneously to glutamate administration and evaluated neuronal degeneration and lesion volume. Representative tissue sections from WT and NOX-2 KO animals stained with cresyl violet and Fluoro-Jade B are shown in Fig. 7a. In the case of WT mice treated with IL-4, we observed a significant decrease in the lesion volume (WT 1.2 ± 0.14 vs WT + IL-4 0.72 ± 0.05 *p* < 0.05; Fig. 7b), as well as a decrease in degenerating cells (WT 82.3 ± 11.1 vs WT + IL-4 35.4 ± 4.7 cells/mm^2^ *p* < 0.05) as compared to mice administered only with glutamate (Fig. 7c).

### NOX activity and caspase-3 activation are regulated by IL-4 in the excitotoxic process

**Fig. 8:**
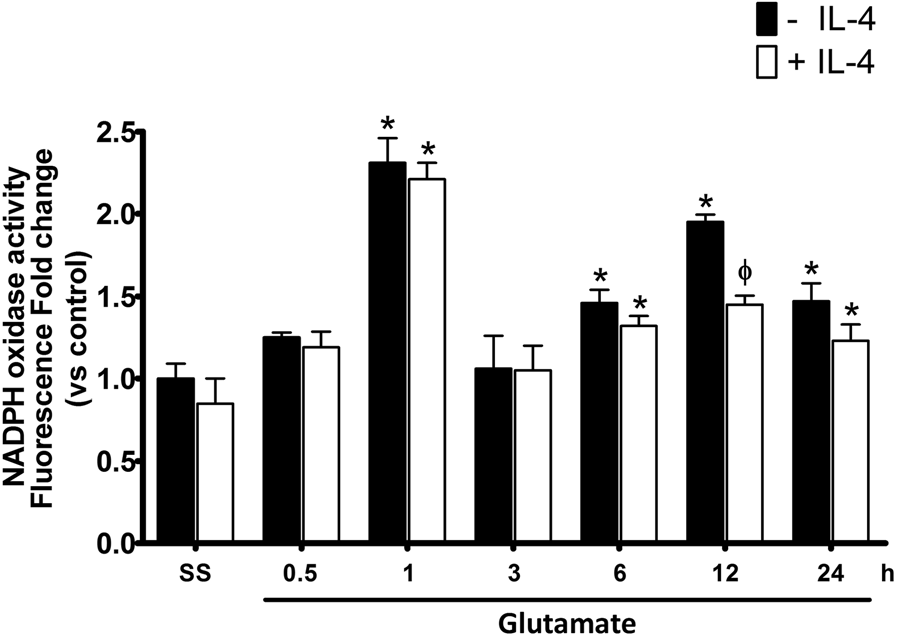
NOX activity in wild type mice treated with IL-4 after glutamate administration. NOX activity was evaluated in WT mice treated with NaCl 0.9% (S.S.), only glutamate (− IL-4) or glutamate + 0.7 ng/mL of interleukin-4 (+IL4); the determination of NOX activity was measured by the fluorescence resulting from the dihydroethidium (DHE) oxidation in striatal homogenates of mice from 0.5 to 24 h (0.5, 1, 3, 6, 12, and 24 h) after glutamate administration. Data are expressed as fold change of fluorescence relative to saline without IL-4. Values are means ± SEM of three independent experiments. *p < 0.05 vs the corresponding saline control; ^ϕ^*p* < 0.05 vs the corresponding time of mice treated with glutamate alone

**Fig. 9:**
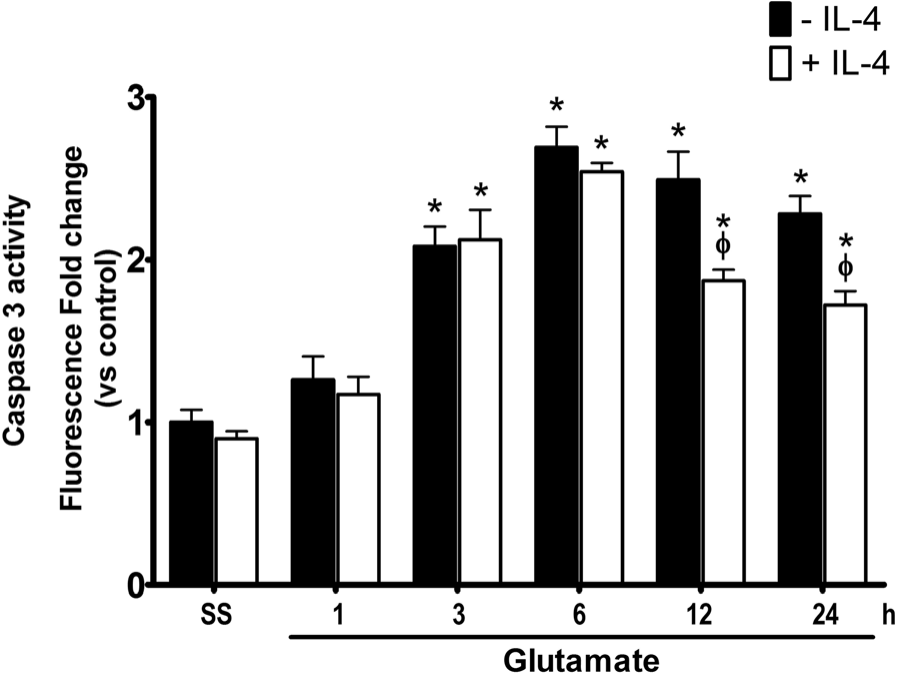
Caspase-3 activity in wild-type mice treated with IL-4 after glutamate administration. Active caspase-3 was measured in striatal homogenates of wild-type (WT) mice treated with NaCl 0.9% (S.S.), only glutamate (− IL-4) or glutamate + 0.7 ng/mL of interleukin-4 (+IL4) after 1, 3, 6, 12, and 24 h. Fluorescence produced by the cleavage of the specific substrate for caspase-3 was quantified as relative units of fluorescence (FRU). Data are expressed as fold change of fluorescence relative to saline without IL-4. Values are means ± SEM of three independent experiments. *p < 0.05 vs the corresponding saline control; ^ϕ^*p* < 0.05 vs the corresponding time of mice treated with glutamate alone

Since the NOX-2 KO mice showed an increased production of IL-4, we explored the possibility of reciprocal regulation through the determination of NOX activity in WT mice treated with IL-4 (Fig. 8). Under these conditions, WT mice treated with IL-4 showed a 50% reduction in NOX activity induced by glutamate only at 12 h (GLU 85 ± 12% and GLU + IL-4 vs 44 ± 12% above control *p* < 0.05; Fig. 8). Concordantly, mice treated with IL-4 also showed a significant reduction in the activation of caspase-3 after 12 h (GLU 249 ± 10% vs GLU + IL-4 187 ± 9% compared with controls, *p* < 0.05) that was maintained at 24 h (GLU 229 ± 9% vs GLU + IL-4 172 ± 10% compared with the control *p* < 0.05; Fig. 9).

### The administration of IL-10 reduces the lesion induced by excitotoxic damage

**Fig. 10:**
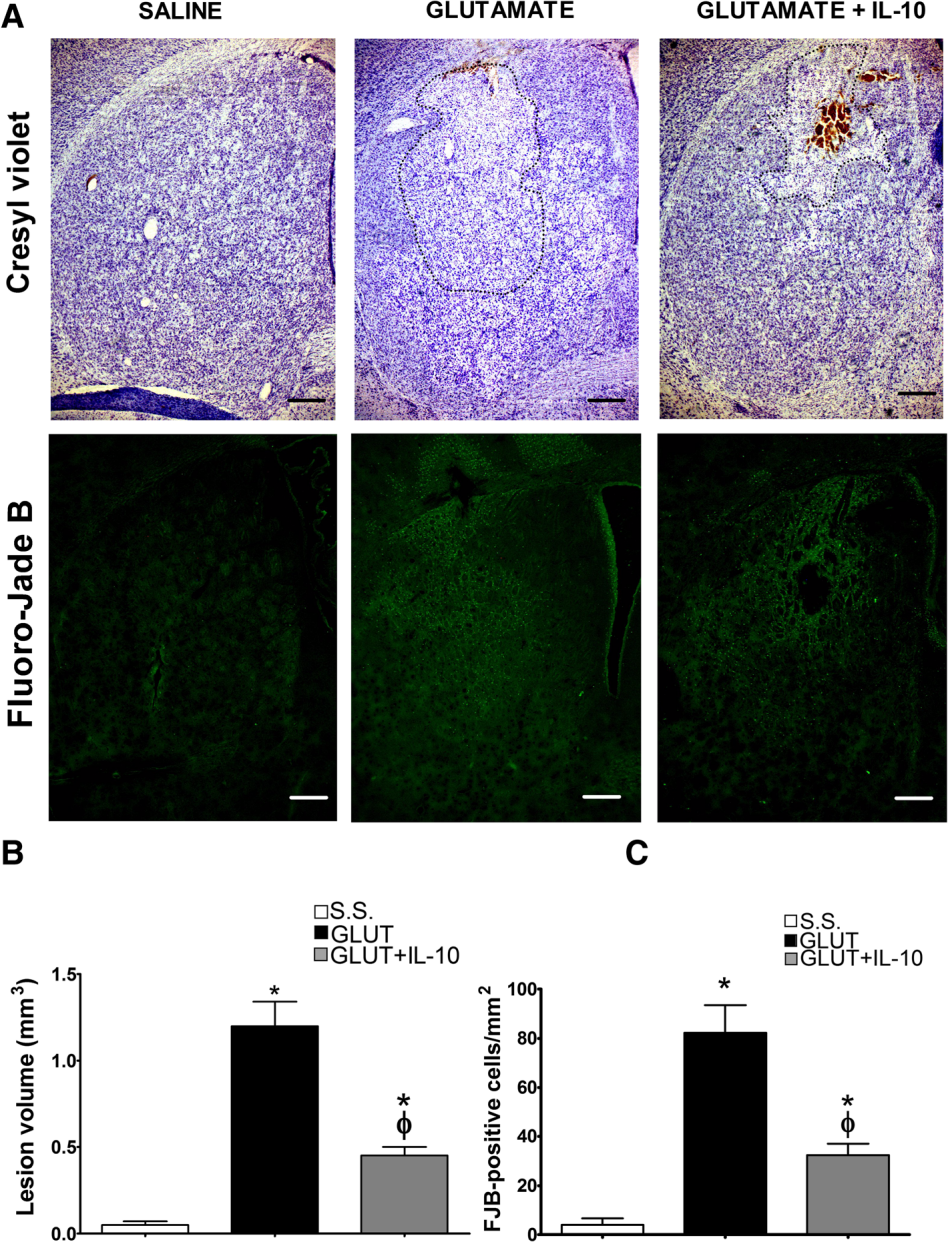
Effect of IL-10 on the lesion area and neuronal degeneration in wild-type mice treated with glutamate. Histological sections of wild-type (WT) mice were obtained after 24 h of intracerebral administration of NaCl 0.9% (S.S.), only glutamate 1 M (GLUT) or glutamate + 0.4 ng/mL of interleukin-10 (GLUT+IL-10) as detailed in the “Methods” section. **a** Representative micrographs of coronal striatal sections showing lesions stained with cresyl violet and damaged cells positive for Fluoro-Jade B. The dotted line delimits the area of lesion of mice treated with glutamate. The scale bars represent 200 μm. **b** Quantification of the lesion volume 24 h after saline or glutamate injection is expressed in cubic millimeters. **c** Total number of FJB-positive cells counted in three slices per mice. Data are expressed as mean ± SEM of six independent experiments. *p < 0.05 vs saline control (S.S.); ^ϕ^*p* < 0.05 vs the corresponding mouse treated only with glutamate (GLUT)

After glutamate administration, we found that the lesion volume was also markedly reduced in mice treated with IL-10. Representative tissue sections from WT and NOX-2 KO animals stained with cresyl violet and Fluoro-Jade B are shown in Fig. 10a. Animals treated with IL-10 showed a decrease of more than 60% in the lesion volume as compared to the untreated mice (GLU 1.2 ± 1.14 vs GLU + L-10 0.45 ± 0.05 mm^3^ *p* < 0.05; Fig. 10b). As for the degenerating cells, the administration of IL-10 also decreased the number of positive cells to Fluoro-Jade B by 60% (GLU 83.4 ± 11.3 vs GLU + IL-10 32.3 ± 4.6 mm^3^ *p* < 0.05; Fig. 10c).

### The NOX activity and caspase-3 activation are regulated negatively by IL-10

**Fig. 11:**
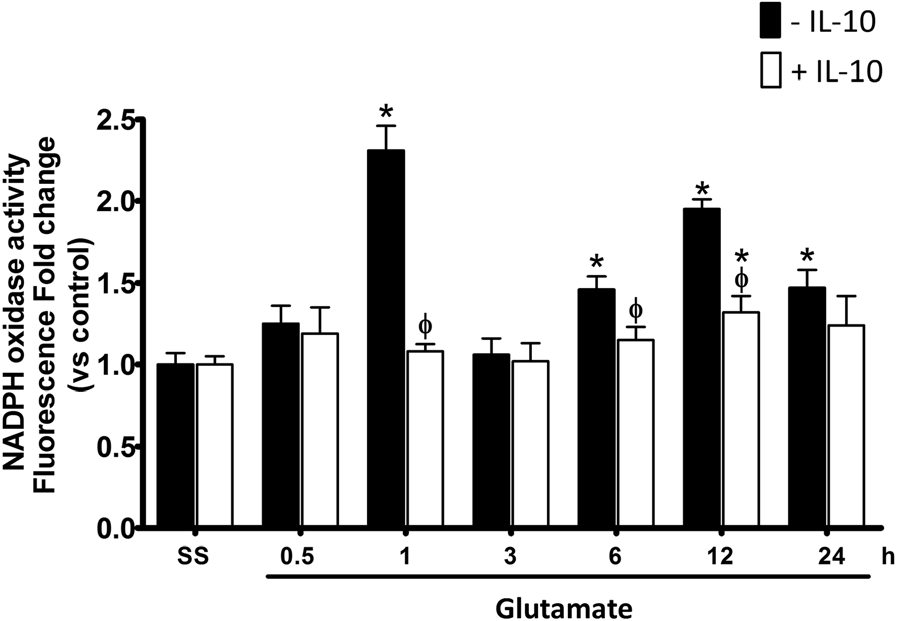
NOX activity in wild-type mice treated with IL-10 after glutamate administration. NOX activity was evaluated in wild-type mice (WT) treated with NaCl 0.9%(S.S.), only glutamate 1 M (− IL-10) or glutamate + 0.4 ng/mL of IL-10 (+ IL-10) as detailed in the “Methods” section. The determination of NOX activity was measured as the change in fluorescence intensity resulting from the dihydroethidium (DHE) oxidation to ethidium (Et) in striatal homogenates after 0.5, 1, 3, 6, 12, and 24 h after glutamate administration. Data are expressed as fold change of Et fluorescence relative to saline without IL-4. Values are means ± SEM of three independent experiments. *p < 0.05 vs the corresponding saline control; ^ϕ^*p* < 0.05 vs the corresponding time of mice treated with glutamate alone

When WT animals were simultaneously treated with glutamate and IL-10, NOX activity was markedly reduced at 1 h (GLU 231 ± 25% vs GLU + IL-10 108 ± 7% of the control, *p* < 0.05); 6 h (GLU 141 ± 5% vs GLU + IL-10 115 ± 4% compared with controls, *p* < 0.05); and 12 h (GLU 195 ± 12% vs GLU + IL-10 132 ± 7% of the control, *p* < 0.05), as compared to mice injected only with glutamate. Nevertheless, 24 h after the excitotoxic insult, both groups showed similar levels of catalytic activity (GLU 147 ± 4% vs GLU + IL-10 134 ± 5% *p* > 0.05; Fig. 11).

**Fig. 12:**
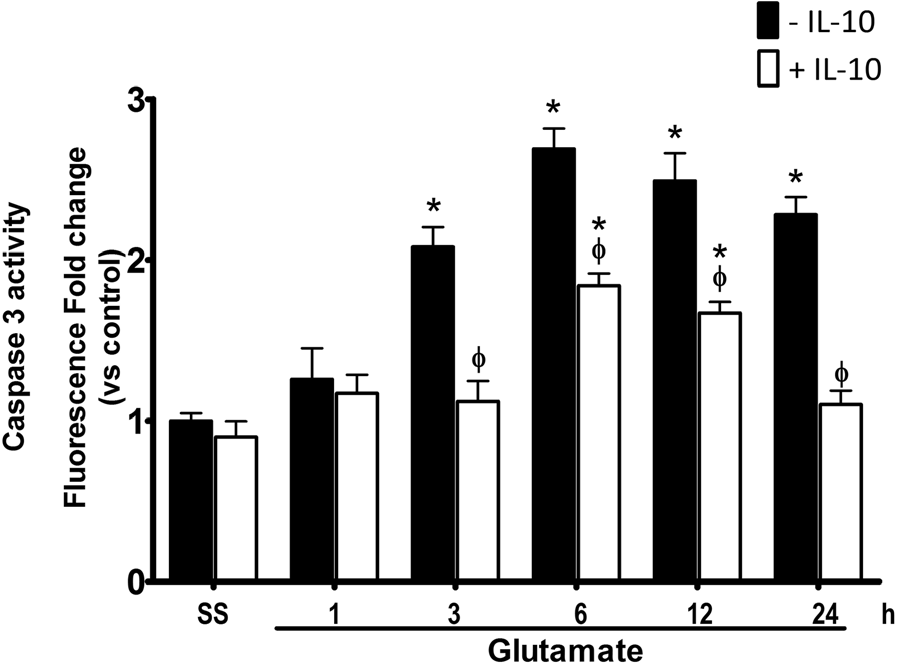
Active caspase-3 in mice treated with IL-10 after glutamate administration. Active caspase-3 was measured in striatal homogenates of wild-type (WT) mice treated with NaCl 0.9% (S.S.), only glutamate 1 M (− IL-10) or glutamate + 0.4 ng/mL of IL-10 (+ IL-10) at 1, 3, 6, 12, and 24 h after glutamate administration. Fluorescence produced by the cleavage of the specific substrate was quantified in relative units of fluorescence (FRU). Data are expressed as fold change of fluorescence relative to saline without IL-4. Values are means ± SEM of three independent experiments. *p < 0.05 vs the corresponding saline control; ^ϕ^*p* < 0.05 vs the corresponding time of mice treated with glutamate alone

Under these conditions, IL-10 also decreased the activity of caspase-3. As we showed previously, the administration of glutamate promotes the increase of active caspase-3 from 3 to up 24 h after the injection. The co-administration of IL-10 reduced caspase-3 activation by 90% at 3 h (GLU 201 ± 18% vs GLU + IL-10 112 ± 4% above control, *p* < 0.05) and 24 h (GLU 228 ± 27% vs GLU + IL-10 118 ± 6% above control, *p* < 0.05) after the injection. At 6 h and 12 h, it decreased approximately 50% (GLU 270 ± 30% vs GLU + IL-10 194 ± 15% of the controls, *p* < 0.05; GLU 249 ± 10% vs GLU + IL-10 177 ± 6% compared with controls, *p* < 0.05; Fig. 12).

### IL-10 administration improves motor recovery in mice after excitotoxic damage

**Fig. 13:**
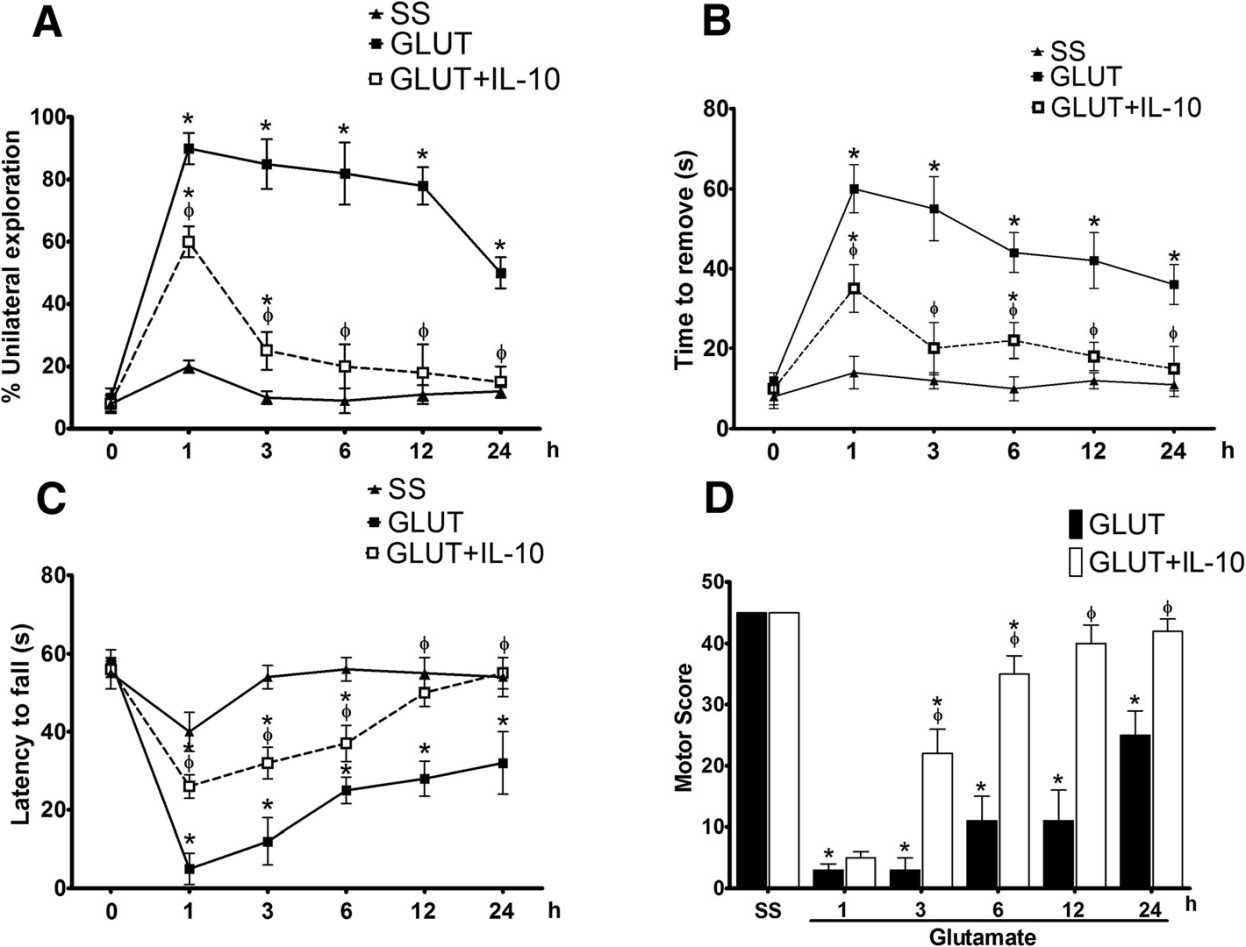
IL-10 improves motor recovery in wild-type mice after glutamate administration. Motor behavior of wild-type mice injected with NaCl 0.9% (SS), 1 M glutamate (GLUT) and glutamate + 0.4 ng/mL of IL-10 (GLUT + IL-10), was evaluated in three tests of motor behavior at 1, 3, 6, 12, and 24 h after glutamate intracerebral injection. **a** Cylinder test, in which each mouse obtained a percentage based on the ratio of unilateral and bilateral explorations performed in three trials, of 3 min each; the percentage was expressed as mean ± SEM of six independent tests. **b** Adhesive removal test quantifies the time in which the animals remove a label previously placed by the examiner. The time of removal was quantified in seconds and was expressed as means ± SEM of six independent tests. **c** Inverted grid test was evaluated by exposing the mice to the inverted grid test in which the decay time was quantified for three non-consecutive opportunities of a maximum of 60 s of duration. The results are expressed as means ± SEM of the latency to fall of six independent tests. **d** Integration of behavioral performance of three previous tests was carried out by assigned an arbitrary score to the performance of each animal, giving the highest score to the performance of the mice injected with saline (45 points). The results are expressed in arbitrary units of motor score as the mean ± SEM of the latency to fall of six independent tests. *p < 0.05 vs the corresponding saline control; ^ϕ^*p* < 0.05 vs corresponding mouse treated with glutamate alone

Mice treated with IL-10 had a better performance in tests of motor behavior after glutamate administration (Fig. 13). In the cylinder test, although mice treated with IL-10 showed a performance similar to those that did not receive treatment, at 1 h (GLU 10 ± 3% vs GLU + IL-10 8 ± 2 unilateral contacts *p* < 0.05) they were able to recover the control level at 6 h (GLU 82 ± 5% vs GLU + IL-10 20 ± 4% unilateral contacts, *p* < 0.05), while this did not happen in the untreated mice (Fig. 13a). In the adhesive test, mice treated with IL-10 were also able to recover baseline performance at 3 h (GLU 55 ± 8 s and GLU + IL-10 20 ± 6.5 s, *p* < 0.05), while those not treated did not reach these recovery levels in 24 h (GLU 36 ± 5 s vs GLU + IL-10 15 ± 5 s, *p* < 0.05; Fig. 13b). In the inverted grid test, mice treated with IL-10 had a better performance than the untreated mice from the first hour after glutamate treatment (GLU 5 ± 4 s vs GLU + IL-10 26 ± 3 s *p* < 0.05). In contrast to the animals treated with glutamate alone, these animals recovered to values similar to the control group after 12 h (SS 58 ± 2 s vs GLU + IL-10 50 ± 4 s, *p* > 0.05) and 24 h (Fig. 13c).

The improvements by IL-10 treatment observed in the individual tests were reflected in the NDS (Fig. 13d). We observed a significant high score in mice treated with IL-10 at 3 h (GLU 3 ± 2 points and GLU + IL-10 22 ± 4 points *p* < 0.05), which progressively improved and reached values similar to those observed in control group at 24 h (GLU 11 ± 5 points and GLU + IL-10 40 ± 3 points *p* < 0.05; Fig. 13d). Mice injected only with glutamate also showed a progressive increase in the motor score, but it was significantly lower of the control values at all times.

## Discussion

In ischemia and brain trauma, neurons lose the ability to control ion homeostasis minutes after the primary injury, resulting in the accumulation of intracellular calcium, cell depolarization, release of glutamate, impaired mitochondrial function, energy failure, and elevated reactive oxygen species production [57–59]. This occurs during the first few minutes-hours after the event and will determine the course of brain damage, including neuroinflammation. For example, the use of NMDA receptor blockers in experimental models of ischemia and trauma markedly reduces neuronal damage and neuroinflammation when administered during the first minutes-hours of insult [60, 61]. Diverse studies postulate that the magnitude of the primary response is associated with the severity of the inflammatory response that can last for months or even years [62, 63]. Therefore, it is necessary to evaluate the processes taking place during the early stages of brain injury, as we did in this study. This could help to define the early clinical intervention that can be decisive in the prognosis.

In animal models, the exogenous administration of glutamate produces an overstimulation of synaptic receptor that leads to oxidative stress and neuronal death. Besides, the excitotoxic damage is closely associated with an inflammatory response [64]. During this process, ROS are key pieces since they participate directly in neuronal death and other parallel physiological processes that are also affected by the excitotoxicity [65, 66].

Several sources of cellular ROS have been involved in the excitotoxic damage; one of the most interesting is the NOX family, whose only known function is ROS production [64, 67]. The seven homologs (NOX-1 to NOX-5 and DUOX-1 and DUOX-2) have different subcellular localizations, and their activation is associated with various signaling pathways of the cell physiology [25, 48]. Particularly, NOX-2 seem to be involved in cell death, since its inhibition markedly reduces neuronal damage in several pathological processes such as ischemia, hypoglycemia, and brain trauma [8, 9, 14], where excitotoxicity is a main component.

In previous studies, we demonstrated that pharmacological inhibition of NOX in primary cultures of cerebellar granule neurons decreases apoptotic death caused by different conditions [67, 68]. Using an animal model of genetic inhibition of NOX-2 or with the administration of NOX inhibitors, we have confirmed the involvement of NOX-2 in excitotoxic neuronal death [13], which correlated with findings of other groups that report that the increase of NOX-2 activity promotes apoptotic death [69].

In the present study, we corroborated that NOX-2 KO mice are less susceptible to excitotoxic damage than WT mice, evidenced by a smaller lesion and less number of positive cells to Fluoro-Jade B in NOX-2 KO mice treated with glutamate (Fig. 2). In this model, we corroborated that NOX-2 is involved in excitotoxic damage since an increase of NOX activity was observed in response to the administration of glutamate. This increase occurred in a biphasic manner (1 and 12 h after glutamate administration). Since both peaks of NOX activity occurred within 12 h of difference (Fig. 3), we suggest that this feature is part of two separate phenomena. We hypothesize that the first increase in ROS participates in a direct response to the extracellular increase of glutamate, while the increase observed at 12 h probably corresponds to the cellular response for the recovery of homeostasis; however, more studies are required to test this possibility.

Our results indicated that the lack of NOX-2 is associated with a minor activation of caspase-3 (Fig. 4), probably resulting from a decrease in apoptotic signaling pathways. It is likely that the neuroprotective effect observed in NOX-2 KO mice results from a decrease of the initial damage and reduced activation of subsequent death signaling pathways. These findings correlate with reports in showing that the increase of NOX-2 activity is associated with the activation of caspase-3 and apoptotic death [68, 69]. It is important to emphasize that the excitotoxic damage includes other mechanisms of cell death (necrosis and autophagy) and that the lack of NOX-2 activity does not completely prevent cell death.

Striatal injuries usually result in impaired sensorimotor function and movement execution [70]. The present results show a clear positive correlation between a reduction of the lesion size and a better recovery of motor function. In both WT and NOX-2 KO mice treated with glutamate, the initial motor function was similarly affected, probably due to the action of glutamate on striatal neuronal circuits. However, this condition improves much faster for the NOX-2 KO animals (Fig. 5) according to the damage observed in both animals (Fig. 2).

The increase in NOX-2 activity is relevant for excitotoxic damage and the inflammatory process. It has been suggested that it contributes, along with various cytokines, to the function impairment and secondary damage orchestrated by microglia [71, 72]. Persistent inflammation can promote secondary tissue injury through excess production of proinflammatory factors, such as TNF-α, IL-1β, and IL-6. These factors promote the polarization of the resident microglia to the M1 phenotype, which in turn causes neuronal death; the sum of these events results in the aggravation of the initial injury and a poor resolution of damage.

In a previous study, we observed that during the excitotoxic damage, the activated microglia in NOX-2 KO mice showed different morphological characteristics as compared to WT animals [13]. In the present work, we observed that NOX-2 mice have a different profile of cytokines production. The major change in the pattern of cytokine production corresponded to the increase in anti-inflammatory cytokines such as IL-4, IL-10, and TGF-β, just a few hours after the initiation of the injury (Fig. 6).

As expected, we observed an increase in the production of IL-1β after 6 and 12 h of glutamate treatment. However, we did not observe any differences between the WT and NOX2-KO mice at any time after glutamate treatment (Fig. 6). This contrasts with the observed results in a model of TBI, where NOX2 deficiency induced a lower expression of IL-1β after the trauma [46]. In addition to NOX2, it has been suggested that mitochondrial ROS also play a key role in the inflammatory process through the NLRP3 inflammasome activation [73]. It is known that the production of IL-1β is regulated enzymatically by the NLRP3 inflammasome [74, 75] and a lower IL-1β secretion is observed in macrophages treated with mitochondrial ROS scavengers [42]. The NLRP3 inflammasome is stimulated by ROS [76], and NOX-derived ROS were initially suggested to be necessary for NLRP3 inflammasome activation [77]. However, in mouse macrophages deficient in NOX, IL-1β production is unaffected [78]. This observation suggests that the mechanisms involved in NLRP3 activation and IL-1β production are dependent on the source of ROS production and the type of insult. Possibly in the present experimental conditions, other sources of ROS rather than NOX are necessary for IL-1β production.

One of the most remarkable findings in this study is that in the NOX-2 KO mice, IL-4 production increases very early during the excitotoxic process, which correlates with previous reports, suggesting that IL-4 contributes to the polarization of the microglia to an M2 phenotype promoting an anti-inflammatory response. Recent evidence shows that neurons that survive a noxious stimulus (i.e., ischemia) respond by increasing the production and release of IL-4 [20]. Although the effects of IL-4 on neuronal survival have not been extensively described, it is known that its increase may promote the release of neurotrophic factors that favor axonal repair and neuronal survival.

The increase of IL-4 in NOX-2 KO mice suggests that NOX-2 participates in the negative regulation of this interleukin. This can directly influence the inflammatory response dynamics, since IL-4 contributes decisively to the acquisition of a microglial anti-inflammatory phenotype producer of IL-10. During excitotoxic damage, the increase of NOX-2 activity correlated temporarily with the decrease of anti-inflammatory cytokines and the increase in inflammatory mediators, which agrees with that reported by other groups who postulate that NOX regulates the production and release of anti-inflammatory cytokines [79, 80].

Similarly, the production of IL-10 is also markedly increased, probably induced by IL-4 [21]. The stimulation of IL-4Rα microglial receptors promotes the activation of several signaling pathways, including the activation of Janus kinase 3/transcription 6 (JAK3/STAT6) and insulin receptor substrate 2 (IRS2). This situation promotes the expression of IL-10 by the nuclear factor kappa-light-chain-enhancer of activated B cells (NFκB) and negatively regulates the production of inflammatory interleukins by the suppressor of cytokine signaling 1 (CS1) [80].

It is known that the binding of IL-10 to its neuronal receptors (IL-10R) activates the JAK2/STAT3 pathway, which reduces oxidative stress and promotes neuronal survival [81]. It is possible that part of the observed decrease in caspase-3 activation and cell death in NOX-2 KO animals could be a consequence of a decreased ROS production, by activation of IL-10R during excitotoxicity.

In our model, a single intracerebral dose of recombinant IL-10 decreased caspase-3 activation in WT mice subjected to excitotoxic damage (Fig. 12), and it is important to note that the concentration of IL-10 administered was similar to that found in the NOX-2 KO mice subjected to the excitotoxic event. Our results showed that the treatment with IL-10 negatively regulates NOX activity (Fig. 11), which could also be associated with the reduction of oxidative stress and therefore less neuronal death.

Interestingly, in contrast to what we observed in NOX-2 KO mice (Fig. 4), the administration of IL-10 did not decrease active caspase-3 induced by glutamate treatment in the initial phase (3 and 6 h), but late in the process (12 and 24 h) (Fig. 9). This suggests that IL-10 could promote neuronal survival only during the secondary phase, being the inflammatory process a probable target. This is in line with the finding that the administration of a single dose of IL-4 markedly reduced the lesion volume (Fig. 7), but the decrease in NOX activity (Fig. 8) and caspase-3 activity (Fig. 9) occurred after 12 and 24 h of glutamate administration.

Based on our findings, including the time course of the different parameters evaluated, we can delimit two phases in the progression of neuronal damage induced by glutamate administration: an initial response, from the onset of administration to the subsequent 6 h and a secondary response, from 6 h and up to 24 h post administration. In WT mice, the initial phase is characterized by an increase in NOX activity and IL-12 production, which promotes a proinflammatory state and severe motor deterioration. During the secondary response, a proinflammatory environment predominates (increased IL-1β, IL-6, and TNF-α production and NOX activity), which promotes apoptotic neuronal death. In contrast, in NOX-2 KO mice, the initial response is characterized by an increased production of IL-4 and IL-10, which probably contributes to a decreased apoptotic death and an improved functional recovery.

Interestingly, when IL-4 was administered to WT mice treated with glutamate, NOX activity and caspase-3 activation were reduced only during the secondary response, probably because its effect depends on the increase of other factors. In support of this, when IL-10 was administered to these animals, NOX activity and caspase-3 activation decreased in both the early and the late phase after glutamate administration.

Altogether, these results lead us to propose that the neuroprotection resulting from the genetic inhibition of NOX-2 could be due, at least partially, to a differential response to excitotoxic damage, which is characterized by an early increased production of anti-inflammatory cytokines. We have shown that the inhibition of NOX-2 activity facilitates the production of anti-inflammatory cytokines, which in turn decreases the injury secondary to excitotoxic damage and improves functional recovery.

## Conclusions

The release of proinflammatory cytokines during the excitotoxic cascade promotes a secondary apoptotic death of neurons that survived the damage. During the excitotoxic process and the subsequent inflammatory response, ROS generated by NOX-2 play a decisive role in the extension of the lesion and consequently in the severity of the functional compromise.

We propose that the neuroprotection resulting from a lack of NOX-2 activity could be due, at least partially, to a differential response against excitotoxic damage, characterized by an increased production of anti-inflammatory cytokines. Although it is necessary to determine the exact mechanism by which NOX-2 participates in the regulation of these cytokines, we suggest that the inhibition of NOX-2 activity facilitates the production of IL-4, which contributes to the production of IL-10.

## Additional file

Additional file 1: **Figure S1.** Expression of gp91phox (NOX-2) in the striatum of wild-type and NOX-2 KO mice after glutamate treatment. (A) Levels of gp91phox (NOX-2) at 1, 6, 12, and 24 h after the intracerebral administration of glutamate (1 M). The results are expressed as fold over the relative intensity in relation to the load control (GAPDH), against time in hours (h). Values represent the average ± SEM of four independent tests. *p < 0.05 vs the corresponding saline control. (B) Western blot of gp91phox (band size 65 kDa) and GAPDH (band size 37 kDa) representative images. (TIF 121140 kb)

## Abbreviations

ANOVA: Analysis of variance
CNS: Central nervous system
DHE: Dihydroethidium
DPI: Diphenyleneiodonium
DUOX: Dual oxidases
ELISA: Enzyme-linked immunosorbent assay
Et: Ethidium
FJB: Fluoro-Jade B
GLU: Glutamate
IL: Interleukin
IL-R: Interleukin receptor
JAK: Janus kinase
NDS: Neurological dysfunction score
NOX: NADPH oxidases
NOX-2: NADPH oxidase-2
ROS: Reactive oxygen species
SEM: Standard error of the mean
SOD: Superoxide dismutase
TGF-β: Transforming growth factor beta
TNF-α: Tumor necrosis factor-α
WT: Wild-type
